# Rethinking the therapeutic misconception: social justice, patient advocacy, and cancer clinical trial recruitment in the US safety net

**DOI:** 10.1186/1472-6939-15-68

**Published:** 2014-09-20

**Authors:** Nancy J Burke

**Affiliations:** 1 Department of Anthropology, History, and Social Medicine, University of California, San Francisco, Box 0128, 1450 3rd Street HD552, PO Box 589001, San Francisco, CA 94158-9001, USA

## Abstract

**Background:**

Approximately 20% of adult cancer patients are eligible to participate in a clinical trial, but only 2.5-9% do so. Accrual is even less for minority and medically underserved populations. As a result, critical life-saving treatments and quality of life services developed from research studies may not address their needs. This study questions the utility of the bioethical concern with therapeutic misconception (TM), a misconception that occurs when research subjects fail to distinguish between clinical research and ordinary treatment, and therefore attribute therapeutic intent to research procedures in the safety net setting. This paper provides ethnographic insight into the ways in which research is discussed and related to standard treatment.

**Methods:**

In the course of two years of ethnographic fieldwork in a safety net hospital, I conducted clinic observations (n = 150 clinic days) and in-depth in-person qualitative interviews with patients (n = 37) and providers (n = 15). I used standard qualitative methods to organize and code resulting fieldnote and interview data.

**Results:**

Findings suggest that TM is limited in relevance for the interdisciplinary context of cancer clinical trial recruitment in the safety net setting. Ethnographic data show the value of the discussions that happen prior to the informed consent, those that introduce the idea of participation in research. These preliminary discussions are elemental especially when recruiting underserved and vulnerable patients for clinical trial participation who are often unfamiliar with medical research and how it relates to medical care. Data also highlight the multiple actors involved in research discussions and the ethics of social justice and patient advocacy they mobilize, suggesting that class, inequality, and dependency influence the forms of ethical engagements in public hospital settings.

**Conclusion:**

On the ground ethics of social justice and patient advocacy are more relevant than TM as guiding ethical principles in the context of ongoing cancer disparities and efforts to diversify clinical trial participation.

## Background

In 1993 the National Institutes of Health passed the NIH Inclusion Act, designed to address the overwhelming reliance on white middle class men for pharmaceutical drug development. Although immediately concerned with the generalizability of treatments, policy changes subsequent to the Inclusion Act presume that participation in clinical trials "produces therapeutic benefits that should be accessible to all" [1,2]. Since this time, a proliferation of research into the how, why, and why not of clinical trial participation and recruitment has led to what Steven Epstein has characterized as a new field of study: recruitment science or recruitmentology [3]. Generally structured according to ethnically defined differences in attitudes toward research–including barriers such as historically-based mistrust in the healthcare system, fears of experimentation, and misunderstandings of scientific methods such as randomization–the recruitmentology literature largely misses, or obfuscates, class and access issues as bases for low participation rates [4].

Importantly, this literature focuses on increasing participation of minority individuals in trials taking place in academic medical centers, more than pharmaceutical industry sponsored trials in private practices and contract research organizations (CROs). This is due to a shift that occurred in the structure of US clinical trials in the 1990s as private sector for-profit research organizations replaced universities in pharmaceutical contracts from more than 80% in the academy prior to 1990 to less than 35% in 2005 [5-7]. Adriana Petryna [8,9], Jill Fisher [6,10], Roberto Abadie [11] and others have chronicled the impact CROs and their effective recruitment strategies have had on underserved and drug naïve populations in the United States and globally. This research has shown that participation in clinical trials taking place in low resource settings often provides the only access available to professional medical care in dilapidated and substandard healthcare systems.

In the United States academic medical centers often serve as both the "safety net" for poor and medically underserved individuals and as sites for innovative medical research. While definitions of safety net institutions vary, the Institute of Medicine’s 2000 report *America*’*s Health Care Safety Net*: *Intact But Endangered* defines "core safety net providers" as those institutions who offer patients access to services regardless of their ability to pay and serve a "substantial share" of uninsured, Medicaid, and other vulnerable patients [12]. In those safety net institutions also serving as teaching and research sites, faculty and providers often receive research funding from the National Institutes of Health or other federal agencies. Since the passage of the NIH Inclusion Act in 1993, all such funded research has been required to actively recruit and include women and minorities. Despite this, underserved cancer patients are typically offered fewer opportunities to participate in research than other patients and recruitment of minority participants remains a substantial challenge [13-16]. As a result, critical life-saving treatments and quality of life services developed from research studies may not address their needs. Therefore, it is important to understand what actually happens when complex cancer clinical trials are discussed with medically underserved and ethnic minority patients in safety net settings.

Parallel but not intersecting with these shifts in the structure and implementation of US scientific research in the in the 1990s has been the emergence of the concern with and study of *therapeutic misconception* (TM) among bioethicists. Defined as "when a research subject fails to appreciate the distinction between the imperatives of clinical research and of ordinary treatment, and therefore inaccurately attributes therapeutic intent to research procedures," [17,18] therapeutic misconception concerns the conflation of research and medical care. According to the definition of TM, true informed consent, and therefore ethical participation, is only possible if patients understand the differing intentions–and roles–of research and medical care staff. Importantly, TM is often studied as a phenomenon among white, middle class, insured populations [17-19]. In addition, the informed consent described in the TM literature is largely limited to discussion of the elements of the informed consent form. Such discussions focus on the risks and benefits of research participation, experimental procedures in the protocol, and the rights of research participants. Not considered in the TM literature are the discussions that happen prior to the informed consent, those that introduce the idea of participation in research. As this paper explores, these preliminary discussions are elemental especially when recruiting underserved and vulnerable patients for clinical trial participation who are often unfamiliar with medical research and how it relates to medical care.

Considerations of health literacy suggest a different way to approach therapeutic misconception as a risk to ethical conduct of research recruitment. The World Health Organization (WHO) Commission on the Social Determinants of Health (2007) defines *health literacy* as "the cognitive and social skills which determine the motivation and ability of individuals to gain access to, understand, and use information in ways which promote and maintain good health" [20]. Being informed in the manner envisioned by bioethicists concerned with TM involves skills and competencies that relate both to the information itself and to the medium used to access that information [21]. Health literacy has recently been linked to the ethical debate regarding equitable access to research in the concept of ‘research literacy,’ "the cognitive and social understanding of the basic purpose, process, and value of research and research participation" [22]. Research literacy, as currently conceptualized, depends upon information and education to address therapeutic misconception [22]. The ethnographic data presented in this paper suggests that considerations of the entire clinical trial communication process, including the initial introduction of the idea of participation in research, should be considered elemental opportunities for addressing (and perhaps assessing) research literacy.

Understanding the different roles of research and medical care staff is especially difficult when the therapeutic and research roles are embodied in the same person, and when this person is the gatekeeper to research participation. Little research has considered the relevance of bioethical concerns about therapeutic misconception to clinical trials recruitment of minority and medically underserved patients. Based in ethnographic observations of clinical trial discussions in public hospital oncology clinics, this study questions the saliency of the therapeutic misconception concept in the recruitment of medically underserved cancer patients for participation in clinical research, and suggests that the on the ground ethical logics of social justice and patient advocacy may be more useful for understanding the perspectives and communicative strategies of differently positioned individuals in the clinic.

This paper reports findings from the ethnographic phase of a study of information disparities in cancer clinical trial recruitment in safety net settings. Framed as a case study, the paper includes in-depth ethnographic description of a cancer clinical trials recruitment discussion to 1) elucidate the ethics employed in clinical trials discussions; 2) describe multiple competing perspectives on appropriate communication; and 3) demonstrate how and when the informed consent process actually begins, and how elemental these preliminary discussions are to patient understanding and acceptance of research participation.

## Methods

This paper draws upon ethnographic research conducted in safety net hospital oncology clinics over the course of two years during which I conducted in-depth interviews with 37 oncology patients in either English or Spanish and with 15 providers (oncologists, surgeons, fellows, residents, patient navigators, social workers, and nurse practitioners). I systematically observed over 150 clinic days, the details of which I recorded in field notes. All participants in the study provided informed consent (verbal during observations; written during interviews). All study procedures were reviewed and approved by the University of California, San Francisco Committee on Human Research.

The combination of interviews and observations provided a complex rendering of the range of experiences of, and communications around, cancer care and clinical trial recruitment. Observations took place in two outpatient oncology clinics in a Northern California safety net hospital from 2008 to 2010. All patients and providers in the clinics were considered eligible for participation. At the beginning of each clinic day, I asked a resident, fellow, or oncologist if I could shadow him/her during clinic and record my observations in a small notebook I kept with me. Throughout the clinic day I followed these providers into the spaces where cases were presented to attendings, pathology reports discussed, eligibility determined, and treatment plans outlined. Prior to each patient visit, I approached the patient and asked if s/he minded if I observed the visit, ensuring her/him in accordance with a verbal consent script approved by the University Committee on Human Subjects Research that refusing to do so would in no way influence the care given. In the course of two years, one patient asked that I not be present due to the presence of her two sisters and the small size of the examination room. Because these clinics took place in a teaching hospital, it was common to have a number of people in the exam room, often times taking notes. Therefore the addition of the anthropologist was less disruptive than it may have been in a different care setting. I asked all patients who were offered participation a clinical trial to participate in an in-person, in-depth interview at a time and place convenient for them. Interview participants signed an informed consent document (translated to Spanish for those who preferred to be interviewed in Spanish) and were given a $30 incentive in appreciation of their time.

In addition to clinic observations and interviews, I attended patient education/support groups, grand rounds, pre-clinic staff meetings, and tumor boards. I used standard qualitative methods, including member checking, to organize and code resulting interview and field note text [23] in order to develop a thematic understanding of oncology practice, clinical trial recruitment communications, and patient experience in the clinics [24]. The findings reported here draw primarily from observations recorded in field notes, but are informed by insights gained through detailed coding and analysis of all interview and field note data.

### The research setting

Scheduling practices and flow differed substantially between the two clinics in which I worked but both were time-pressured environments with overworked and very committed staff. At times the atmospheres were chaotic with people rushing from place to place, addressing multiple emergencies at once. At others there were lulls during which providers discussed having attended a patient’s funeral over the weekend, or having checked in on a recently admitted patient whose cancer treatment had caused complications, generally expressing their care and concern for patients beyond the clinic.

In the general oncology clinic, patients were assigned a consistent provider, usually an oncology fellow, for up to six months, at which point fellows rotated out of the clinic. Patients received a specific appointment date and time linked to their provider’s schedule. Despite this, patients waited one to four hours in the clinic waiting room. The hallways were often filled with patients waiting to have their lab tests taken and to meet with social workers. Research assistants recruiting for various projects also clogged the hallways. Staff pulled chairs out of exam rooms for older patients suffering the effects of chemotherapy to sit in, especially those uncomfortable with the smells and discussions occurring in the over crowded waiting room. The walls in the hallways were adorned with black and white photographs of cancer patients, some recently deceased, who had participated in the hospital’s support group program.

The interdisciplinary breast clinic was held in another part of the hospital one afternoon a week. Unlike the general oncology clinic, patients were scheduled in blocks in breast clinic, resulting in waiting rooms that filled at the beginning and midway through the afternoon, and wait times were similar to those of the general oncology clinic: between one and four hours. Unlike the general oncology clinic, the breast clinic walls were painted an off white and left unadorned except for a white board reporting provider vacations. Chairs were not allowed in the hallway and patient navigators (bilingual staff providing informational and practical support to guide patients through interdisciplinary breast cancer treatment), medical interpreters, the clinical trials coordinator, and researchers leaned against walls waiting for appointments and patient arrivals. Patients never knew which provider they would see in this clinic, unlike the general oncology clinic. Their charts were stacked in order of appointment on a table in the middle of a long hallway. During clinic providers picked up the top chart and reviewed it quickly before going in to see the patient. If the patient was eligible, or thought to be eligible, for a clinical trial, the clinical trials coordinator (CRC) would put a bright sticky note on the chart asking the provider to talk with the coordinator before going in to see the patient. Busy surgeons and students and residents rotating through the clinic often avoided these charts and instead selected the next in line. Those who did pick up the charts with a sticky note received a quick description of the trial from the CRC in the hallway outside the exam room while patients and providers streamed by. The provider was tasked with remembering, from this quick description in the hallway, the basics of the trial enough to describe it to the patient prior to inviting the CRC in to administer informed consent.

Breast and general oncology clinic patients range in age, ethnicity, and educational background. The safety net hospital within which these clinics reside serves a diverse patient population of 2.5 million patients a year, 29% of whom are Latino. Patients at this hospital speak approximately 20 different languages. About 80% of the total population that the hospital serves receives publicly funded health insurance (Medicare or Medi-Cal) or is uninsured. Importantly, cancer patients in this safety net hospital tend to be younger, have multiple co-morbidities including substance abuse and mental health issues, and many are non-English speakers or Low English Proficient.

## Results

Interviews revealed that patients often did not remember being offered participation in a clinical trial. All treatment discussions, whether experimental or standard, seemed to stream together in their minds as they recounted struggles to keep up with information, appointments, bills, families and jobs. Patients at this safety net hospital generally, and hence many interviewees, lived in single occupancy hotels or other substandard housing, lacked health insurance, often spoke little English, and were usually grateful for the care they had received since their diagnosis. Many recounted financial difficulties including the need to pay rent, hold down a job while managing appointment scheduling and exhaustion from therapy, and pressure to send remittances to family in countries of origin. Stress stemming from unstable living situations and multiple family responsibilities including parenting challenges were also common themes. In light of these many competing priorities, the expectation that patients acquire the specialist knowledge necessary to recount and make assessments of research versus treatment, the basis for informed consent to participate in research, was particularly challenging.

One of the oncologists in the clinic (Dr. Lee)^a^ staunchly believed this, and invoked a social justice ethic of inclusion and research access to explain her approach to clinical trial recruitment. She stated, "It’s a matter of justice. We need patients like ours represented in these studies. It’s our job to make sure they know about studies and how important it is for them to participate." In response to my comment that few patients interviewed remembered being offered a trial, she said, "ah, that means we’re doing our job." In other words, clinic providers were making research participation a normal part of cancer care. Dr. Lee expressed her strong belief that clinic patients should be represented in the production of evidence and treatment innovation to new oncology fellows when they rotated onto the service. She also discussed the importance of clear communication about clinical trials and the need to be aware of differing literacy levels and cultural orientations. When discussing research participation with breast cancer patients, she explained that the way we know how to provide treatment is because other women participated in early studies, thus invoking a ‘public good’ or ‘gift to other women’ rationale.

In the following I describe a consultation this oncologist had with a monolingual Spanish-speaking patient regarding clinical trial participation. The excerpt, similar to other trial recruitment discussions I observed, highlights challenges to clear communication, differing perspectives on appropriate information, and struggles to both conflate and distinguish research and care. It also highlights the role of actors not present in bioethical debates about therapeutic misconception–but present in the care of underserved patients–and their potential influences on clinical trial recruitment and decision-making. In this safety net hospital, additional influential actors include medical interpreters and patient navigators. Lastly, this field note excerpt illustrates the importance of expanding notions of ethical consent beyond discussion of the consent form to the initial introduction of the possibility of research participation.

### Ethnographic Field note

*It was a busy day in clinic. There were few providers* – *an expected Fellow failed to arrive* – *and a stack of charts with patients waiting in the waiting room. Dr. Lee*, *seated at a desk in the middle of the hallway*, *discussed Millie*’*s chart with the resident who had been in to see her to determine if she was eligible for an ongoing chemotherapy trial. Since this was a teaching clinic*, *small exam rooms often accommodated students and attending physicians as well as patients and*, *sometimes*, *family members or patient navigators. This crowd sometimes increased when trials were to be discussed*, *contributing a feeling of gravitas to the discussion. In this case*, *Millie and her brother watched as Dr. Lee*, *the navigator*, *the clinical trials coordinator*, *the resident*, *and the anthropologist all filed in*.

*Dr. Lee started off explaining that Millie needed to start chemotherapy. She then said that one of the things she wanted to talk about with Millie was the possibility of participating in a study on how best to give chemotherapy. Dr. Lee explained that one of the ways they can find out the best way to give chemotherapy is by asking women with breast cancer to help them figure it out. Millie asked*, "*What do they do in those studies*?" *The navigator interpreted as Dr. Lee explained that this particular study was a chemotherapy study that looks at a couple of old drugs*, *plus one new drug*; *half of the women get the* "*regular stuff*" *and the other half get the additional knew drug. The navigator stopped interpreting*, *turned to Dr. Lee and asked*, "*Is this a randomized trial*"? *Dr. Lee turned to the navigator and responded* "*yes*." *The navigator translated this response as* "*we wouldn*’*t know if you*…*which one you would get because it is basically a lottery*." *Dr. Lee turned to the navigator and asked*, "*How did you say that exactly*?" *Her tone indicated annoyance despite the smile on her face. The resident*, *who also spoke Spanish*, *answered that the navigator had said*, "*It is basically a lottery*." "*The point is*," *Dr. Lee said*, "*You don*’*t decide*, *I don*’*t decide. The computer decides who gets which*; *that*’*s in order for it to be completely fair*." *The resident translated this response. Millie then asked* "*But the two medicines that they gave before worked*?" *The resident translated again. Dr. Lee answered* "*yes*." *Millie started to ask a question regarding the number of medications she would receive. Dr. Lee interrupted*, *saying that the new medication also works*, *because it has been tested in women with breast cancer before. The navigator interpreted*, *saying*, "*It would only be three medications*, *two are the normal medication plus the new medication which they have tested by itself and it has proven to work*, *but they want to know if it works best*…" *Dr. Lee said*, "*We haven*’*t described the trial quite correctly*, *but that*’*s okay*," *to which the navigator responded that Millie was asking if she was going to get the two plus three more medications. Dr. Lee saidno*, *that the medication in both cases were known to work*, *and one of the questions they have is which one works better. The navigator responded that she was trying to explain that to Millie*, *but Millie wanted to know if she would receive more than three medications*, *to which Dr. Lee responded* "*no*." *The navigator interpreted*, "*It will always be the two that they offered before*." *The doctor said*, "*That is the part where you are not quite right*, *but that*’*s okay*." *The navigator asked for clarification*, *to which Dr. Lee responded that it was not important to explain it*; *what was important was to say that in both cases the medicines are known to work and the only question is which one works better than the other. Dr. Lee said*, "*don*’*t discuss the numbers*." *The navigator responded*, "*but she wants to know the number*, *if there are going to be three or five medications*." "*There are no more than three*," *Dr. Lee said*.

*Millie asked* "*The two that work*, *they add one more*, *isn*’*t that too strong for the person*?" *Dr. Lee answered*, "*It is not about muy fuerte*" *and added that the secondary effects are different*, *but about the same. Millie asked*, "*When they give three medicines*, *is the dose less*?" *The resident again interjected*, *stating that the doses have been established in previous parts of the experiment. The navigator responded*, *but* "*she wanted to know if she is going to get the same number of doses*," *to which Dr. Lee responded that this was more detail than was necessary at this point*; *what they really wanted to ask was if Millie would think about the possibility of being part of the study*, *and that the details would be explained later if she decided to do it. The question was about the principle of whether or not she would be interested in being part of a research study*.

## Discussion

The competing logics of clinical trial recruitment and immediate concerns and needs [25] cause a rupture in management of the discussion in my field notes. Whereas Millie was focused on immediate bodily therapeutic concerns (numbers of drugs, intensity of dosages, and variability of side effects), Dr. Lee asked her to push these concerns aside to consider the abstract idea of participating in research. The consideration of research participation, therefore, was taken somewhat out of the treatment context, despite Dr. Lee’s desire to manage research as a normative part of treatment. The confusion inherent in these competing logics became clear later in the conversation when Millie seemed to come to an understanding of the similarity between the proposed study and standard care and stated, "so I don’t need to make a decision." Assured by the navigator that she actually did need to, Millie reasserted her need to know "all the details" before doing so.

Some argue that the lack of improvement in terms of decreasing mortality due to cancer since the institution of Nixon’s "War on Cancer" over 40 years ago is due to the low participation rate–variably reported as 2 to 9% - of eligible adult oncology patients in studies of cancer treatments. A 2010 *JAMA* commentary noted that the 2010 Institute of Medicine Report (*Transforming Clinical Research in the United States*) and initiatives such as the Clinical Trials Transformation Initiative "recognize that successful recruitment and retention of participants in clinical trials is critical for improving the efficiency and effectiveness of phase III and IV clinical trials and that action to ensure adequate enrollment is urgently needed" [26]. The authors go on to state that "the importance of health care professionals as gatekeepers for clinical trial participation cannot be overstated" [26].

The question of who is considered a gatekeeper in clinical trials recruitment is an important one raised in my research. In much of the bioethics and public health literature on informed consent and ethical participation in research, the "problem" is framed as lack of clarity in information, rather than equity in collaboration or access. "Information," in this sense, is understood as a necessary precursor to the development of new partnership and shared decision-making relationships between healthcare practitioners and patients [21]. However, this model of patient education tends to assume a binary exchange between provider and patient, without accounting for the presence of others who might introduce competing ethics and understandings based in patient advocacy into the discussion. By introducing the concept of randomization into Dr. Lee’s presentation of the research design, the patient navigator signaled an essential difference between the clinical trial and standard care. She also advocated her own version of social justice by influencing the direction of the conversation toward clarifying and discussing these differences, rather than directly interpreting what was being said.

Elsewhere, I have discussed the challenges evident in this communication as clinic "noise" [27]. In other words, providers may receive patient questions about treatment, doses, and bodily effects as obstructive "noise" complicating the clinical trial discussion, while patients perceive these same questions as essential information needs stemming from concerns about time away from work, energy for parenting, and other living conditions. Patients, in this shared-decision-making framework, are tasked with understanding the differences between the scientific model of research and medical care, with relatively little, even sometimes conflicting, guidance on how to do that. If unable to do so, they suffer from misconception. In this model potential research participants are produced as bearers of both rights and responsibilities. They have entitlements to disclosure, but obligations to assess the information they are given carefully, to understand, to make rational decisions, and to avoid false hope or expectations [28]. Clinicians, too, emerge as having rights and responsibilities. They have the right to solicit agreement and study participation from their patients and also the responsibilities to ensure patients continue to receive proper therapeutic care, to discuss clearly and completely when and why treatment and research protocols deviate, and to avoid inducing false hopes or expectations. Importantly, the discussion in my field note, like many I observed, took place prior to the discussion usually considered in ethical analyses of clinical trial recruitment. One could argue that this field note records a discussion of assent to discuss the possibility of participating, and therefore is not subject to the concerns raised by bioethicists regarding therapeutic misconception. At the same time, this field note demonstrates how and when the informed consent process actually begins, and how elemental these preliminary discussions are to patient understanding and acceptance of research.

Hallowel and colleagues [29] suggest the problem of ethical consent to research participation is the result of the desire to dichotomize and classify what are intrinsically hybrid activities (i.e. activities that might have scientific intent but therapeutic benefit) into either care or research [29]. Rather, they suggest a focus on *what* is going to happen, rather than the *why*[29]. As the conversation between Millie, the navigator, the resident, and Dr. Lee illustrates, slippage between explaining the what and the why of research may cause confusion, tension, and possible mistrust of research. In this case, Millie decided not to participate.

### Limitations

As an inductive, ethnographic study, this research is limited to a recounting of phenomena that occurred at a specific time and in a specific context. While data presented provide insights into communication processes likely relevant to other public hospital settings, the observations were limited to two oncology clinics in one safety net hospital and therefore are not generalizable.

## Conclusions

Bioethicists promote the "ideal clinical trial participant as an autonomous rational volunteer who is active in the consumption of information about his or her health, has options for care outside clinical research, and is able to differentiate and choose among these options given his or her personal health needs" [30]. In other words, the onus is on the patient to comprehend biomedicine rather than on the clinician researcher to understand structural inequalities inherent in US healthcare that constrain patient interactions with the healthcare system and their treatment decision-making. As one prominent bioethicist, and original author of the ‘therapeutic misconception’ paper stated, "most clinical trial participants are middle class people with insurance," [19] thus signifying the class and resources presumed in therapeutic misconception debates. These debates and concerns about persistent therapeutic misconception have strongly influenced the shape of the current bioethics regulatory system in the United States, embodied in local IRBs, the National Bioethics Advisory Commission (NBAC), federal mandates, and the US Common Rule (45 CFR46). The continuing relevance of the ethical principal of therapeutic misconception for shaping the participation of the increasingly diverse and largely under-insured US populations in clinical research, however, is not clear. As evidenced in recruitment discussions in my research site, the emergence of other ethical principles on the ground, such as those concerned with social justice and patient advocacy, suggest that class, inequality, and dependency influence the forms of ethical engagements in public hospital settings.

## Endnote

^a^All names have been changed to protect the anonymity of participants.

## Competing interests

The author declares that she has no competing interests.

## Author’s contribution

The author conceived of the study, conducted the field research, analyzed the data, and wrote the manuscript.

## Pre-publication history

The pre-publication history for this paper can be accessed here:

http://www.biomedcentral.com/1472-6939/15/68/prepub
